# Senescence Accelerates the Occurrence of Dual Amyloidosis: Alzheimer's Disease and Wild-Type Transthyretin Amyloidosis

**DOI:** 10.7759/cureus.81753

**Published:** 2025-04-05

**Authors:** Yohei Misumi, Yukio Ando, Naoya Nakashima, Yusuke Sugimura, Ryo Shirahama, Ryo Noguchi, Hirofumi Matsuda, Keiko Ando, Naoko Tsunoda, Yasuhiro Izumiya, Kenichi Tsujita, Mitsuharu Ueda

**Affiliations:** 1 Department of Neurology, Kumamoto University, Kumamoto, JPN; 2 Department of Neurology, Amyloidosis Supporting Center, Sugimura Hospital, Kumamoto, JPN; 3 Division of Cardiology, Sugimura Hospital, Kumamoto, JPN; 4 Division of Neurology, Sugimura Hospital, Kumamoto, JPN; 5 Division of Gastroenterology, Sugimura Hospital, Kumamoto, JPN; 6 Department of Psychiatry, Mitsugumachi Clinic, Kumamoto, JPN; 7 Department of Cardiovascular Medicine, Kumamoto University, Kumamoto, JPN

**Keywords:** alzheimer’s disease, dementia, dual amyloidosis, heart failure, transthyretin amyloidosis

## Abstract

We report a case of dual amyloidosis with Alzheimer's disease and wild-type transthyretin (ATTRwt) amyloidosis. A 76-year-old man with Alzheimer's disease was referred for anti-amyloid-β therapy with lecanemab. He also had symptoms of congestive heart failure and a history of carpal tunnel syndrome, cubital tunnel syndrome, and lumbar spinal stenosis; raising Technetium-99m pyrophosphate myocardial scintigraphy showed abnormal uptake, and histopathologic examination revealed transthyretin (TTR) amyloid deposition in both myocardial and gastrointestinal biopsy specimens. Genetic testing for the *TTR* gene revealed no variants. The diagnosis of ATTRwt amyloidosis was confirmed, and treatment with a TTR tetramer stabilizer, tafamidis, was initiated. Alzheimer's disease of the brain and ATTRwt amyloidosis of the heart are both representative amyloidoses associated with aging. To date, there are no reported cases of dual amyloidosis other than autopsy cases, but considering the high prevalence of both diseases, it is plausible that a significant number of elderly individuals may suffer from both diseases simultaneously but are underdiagnosed. In recent years, disease-modifying drugs effective against both diseases have become available, making early diagnosis increasingly important.

## Introduction

Wild-type transthyretin (ATTRwt) amyloidosis is a major systemic amyloidosis characterized by the aggregation of misfolded wild-type transthyretin (TTR) proteins to form amyloid fibrils. Amyloid deposition in tendon and ligament tissue causes entrapment neuropathy, while amyloid deposition in myocardial tissue causes restrictive cardiomyopathy and arrhythmias. In particular, cardiac involvement is a critical determinant of life expectancy, and the median survival time after diagnosis is three to five years without disease-modifying therapies (DMTs) [1]. ATTRwt amyloidosis predominantly affects older individuals, and its prevalence increases with age [2]. Although its prevalence has been underestimated, autopsy studies have shown that ATTRwt amyloid deposits are present in 25% of Western individuals and 12% of Japanese individuals aged 80 years or older [3,4]. Therefore, the actual number of cases may be significantly high.

Alzheimer's disease (AD), classified as localized amyloidosis in which amyloid β (Aβ) forms senile plaques in the brain, is the most common cause of dementia, accounting for over 60% of cases. According to the World Alzheimer Report 2021, the global prevalence of dementia exceeded 55 million people [5]. AD is a prototypical aging-associated disease, with incidence increasing significantly with age. The life expectancy of individuals with AD is reported to be around eight years [6], during which the associated social and economic burden is substantial.

Given the prevalence of these two forms of amyloidosis (AD in the brain and ATTRwt amyloidosis in the heart), it is plausible that a significant number of elderly individuals may suffer from both diseases simultaneously. However, such cases of dual amyloidosis have rarely been documented in the literature.

Here, we report a case of dual amyloidosis with AD and ATTRwt amyloidosis, a condition that may have been overlooked despite its potential frequency.

## Case presentation

**Figure 1:**
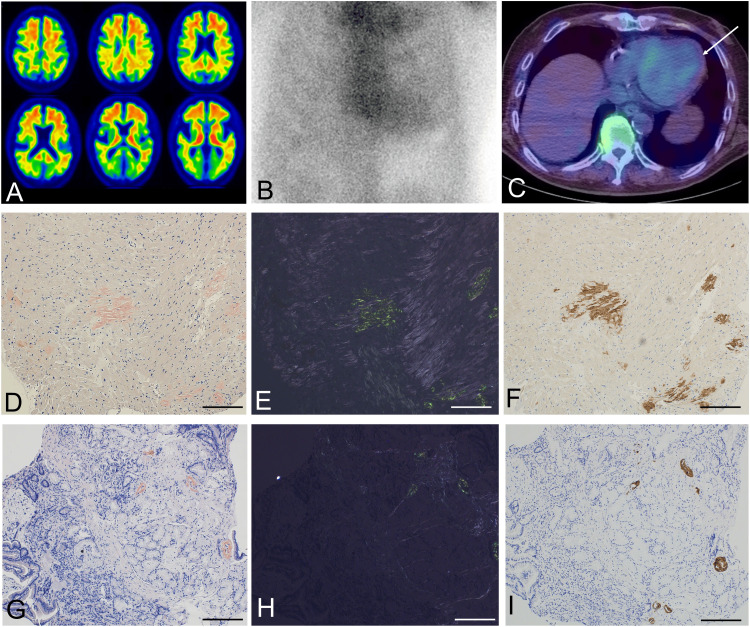
Nuclear medicine exams and pathology images (A) 18F-flutemetamol amyloid PET imaging demonstrates significant amyloid accumulation in the brain. Technetium-99m pyrophosphate (PYP) scintigraphy shows myocardial uptake in (B) planar and (C) SPECT imaging. Congo red staining and immunohistochemistry of biopsied specimens: (D–F) myocardium; (G–I) duodenum; (D, G) Congo red staining observed under bright field; (E, H) Congo red staining observed under polarized light; (F, I) transthyretin immunohistochemistry. Scale bars = 200 μm.

The patient was a 76-year-old Japanese man. He had a history of type Ⅱ diabetes mellitus, hypertension, myocardial infarction, and atrial fibrillation. Twenty years prior, he had undergone surgery for bilateral carpal tunnel syndrome, followed by surgery for right cubital tunnel syndrome 15 years ago and lumbar spinal canal stenosis 10 years ago. Since the age of 66, he has occasionally exhibited repetitive speech and questioning behaviors. His recent memory impairment progressed very slowly, and at the age of 70, he visited a psychiatrist and was diagnosed with AD. At the age of 76, he was referred to our hospital for treatment with the newly approved anti-Aβ antibody lecanemab. Cognitive impairment was characterized mainly by recent memory impairment, with a Mini-Mental State Examination score of 23 and a Clinical Dementia Rating of 1.0. 18F-flutemetamol amyloid PET imaging showed significant accumulation in the bilateral frontal, parietal, basal ganglia, and posterior cingulate gyrus-precuneus areas (Figure 1A), indicating the presence of AD pathology. Additionally, a brain MRI revealed mild hippocampal atrophy, supporting the diagnosis of AD. Clinically silent multiple lacunar infarcts were identified in the basal ganglia region, but there were no significant microbleeds on susceptibility-weighted imaging. Treatment with lecanemab was initiated.

In this case, coexisting ATTRwt amyloidosis was suspected due to the history of recurrent tendon and ligament disorders such as bilateral carpal tunnel syndrome, atrial fibrillation, and NYHA (New York Heart Association) grade II heart failure symptoms. In ATTRwt amyloidosis, amyloid deposition in tendon and ligament tissue causes entrapment neuropathy, and subsequent amyloid deposition in myocardial tissue causes restrictive cardiomyopathy and arrhythmias. Echocardiography revealed no significant wall thickening, but a septal E/e' was suggestive of diastolic dysfunction. Chest X-ray revealed cardiac enlargement. N-terminal pro-B-type natriuretic peptide (NT-proBNP) levels and high-sensitivity troponin T (hs-TnT) levels, which correlate with the severity and prognosis of ATTRwt amyloidosis, were elevated (Table 1). No M-protein was detected, and free light chains were not elevated. The genotype of apolipoprotein E was ε4/ε4. Technetium-99m pyrophosphate (PYP) myocardial scintigraphy revealed grade 2 abnormal uptake (Figures 1B, 1C), consistent with ATTRwt amyloidosis. Genetic testing for the *TTR* gene showed no variants. Histopathological examination using Congo red staining and immunohistochemistry revealed TTR amyloid deposits both in myocardial and gastrointestinal biopsy specimens, demonstrating systemic amyloidosis (Figures 1D-I). A diagnosis of ATTRwt amyloidosis was confirmed, and treatment with a TTR tetramer stabilizer, tafamidis, was initiated. Regular follow-up over six months showed no significant progression of cardiac amyloidosis.

**Table 1:** Blood test findings AST: Aspartate aminotransferase, ALT: Alanine aminotransferase, CPK: Creatine phosphokinase, Cr: Creatinine, BUN: Blood urea nitrogen, HbA1c: Hemoglobin A1c, CRP: C-reactive protein, BNP: B-type natriuretic peptide, NT-pro BNP: N-terminal pro-B-type Natriuretic Peptide, hs-TnT: high-sensitivity Troponin T.

Laboratory Test	Result	Reference Range
White Blood Cell Count (×10³/μL)	7.7	4.0-9.0
Hemoglobin (g/dL)	12.9	13.0-18.0
Platelet Count (×10^3^/μL)	156	120-380
Albumin (g/dL)	4.0	4.1-5.1
AST (U/L)	23	13-34
ALT (U/L)	24	7-37
CPK (U/L)	82	57-284
Total Cholesterol (mg/dL)	137	128-220
Triglyceride (mg/dL)	276	30-150
Cr (mg/dL)	1.13	0.60-1.20
BUN (mg/dL)	29.7	8.4-21
HbA1c (%)	6.7	4.6-6.2
CRP (mg/dL)	0.01	0-0.3
BNP (pg/mL)	241.5	0-18.4
NT-pro BNP (pg/mL)	1770	0-59
hs-TnT (ng/mL)	0.025	0-0.014
M-protein	Negative	Negative

## Discussion

The approval of lecanemab, an anti-Aβ antibody drug, in Japan in 2023 has ushered in a new era in the treatment of AD [7]. In addition, more accurate early diagnosis using amyloid PET and cerebrospinal fluid biomarkers is now required to implement these therapeutic interventions.

Similarly, in ATTRwt amyloidosis, the recent development of DMTs such as TTR stabilizers and gene silencers has significantly improved the prognosis of this long-untreatable disease [8,9]. Although the long-term therapeutic effects of DMTs have not been thoroughly analyzed, Kuyama et al. reported that two advanced ATTRwt patients treated with tafamidis, a TTR stabilizer, did not progress to cardiac dysfunction for more than nine years [10]. Vutrisiran, a gene silencer, was approved by the FDA in 2025 for the treatment of TTR-mediated amyloid cardiomyopathy.

Both in hereditary and wild-type ATTR amyloidosis, the earlier DMTs are started, the better the therapeutic response and prognosis. Importantly, it is well documented that carpal tunnel syndrome, cubital tunnel syndrome, shoulder rotator cuff tears, and spinal canal stenosis due to ATTRwt amyloid deposition often occur around 10 years before the development of cardiac amyloidosis [11,12]. These orthopedic histories provide clues to suspect cardiac amyloidosis due to ATTRwt amyloid deposition as described in the cardiac amyloidosis guidelines [13].

Regarding the incidence of these two types of amyloidosis, a significant number of elderly individuals may have both types of amyloidosis simultaneously, but the reported cases to date are limited [14,15]. In an analysis of 63 autopsies of Finns aged 95 years and older, 23 of 63 (37%) had ATTRwt amyloidosis, of whom eight had a clinical diagnosis of AD, but no association was found between ATTRwt amyloidosis and the incidence of neuropathologically defined AD [14].

The reason for the coexistence of the two types of amyloidosis in our case is unknown. The genotype of apolipoprotein E in this patient was homozygous ε4, a risk factor for AD, but the effect of this genotype on ATTRwt amyloid deposition has not been demonstrated. In patients with ε4 homozygotes, lecanemab has been shown to reduce Aβ accumulation in the brain, but the risk of amyloid-related imaging abnormalities (ARIA) is significantly higher [7]. After obtaining informed consent, we administered lecanemab to the patient and performed a careful MRI follow-up. TTR, which is also produced in the choroid plexus, has been reported to act as a protective protein against Aβ amyloid formation by binding to Aβ, promoting Aβ clearance from the brain, and inhibiting Aβ aggregation [16]. However, it is unclear whether ATTRwt amyloid formation in the heart affects Aβ amyloid plaque formation in the central nervous system. Given the high prevalence of both diseases in the elderly, this case may be considered a coincidental co-occurrence of the two diseases.

The 21st century is an era of increasing age-related diseases. AD and ATTRwt amyloidosis are representative diseases and have received increasing attention due to the prognostic improvements that can be achieved with the development of new DMTs. Dual amyloidosis of both diseases may be an overlooked disease due to differences in disease areas despite its high prevalence. In particular, cardiomyopathy in ATTRwt amyloidosis progresses insidiously and is often underdiagnosed due to a lack of recognition in clinical practice.

AD is often associated with heart failure [17], and reduced cerebral blood flow, correlating with an increased prevalence of cognitive dysfunction, has been observed in patients with heart failure. Therefore, it is important to screen for heart failure in patients with AD. In addition, in patients with heart failure and a history of multiple orthopedic surgeries, such as bilateral carpal tunnel syndrome, as in our case, it is important to perform bone scintigraphy to diagnose ATTR amyloidosis. Our case is didactic in a recent aging society and draws attention to the possibility of the coexistence of two major forms of amyloidosis, brain and heart, in an era when DMTs are now available for each disease.

## Conclusions

We report a case of dual amyloidosis with AD and ATTRwt amyloidosis. Our case illustrates that multiple amyloidoses due to different amyloid precursor proteins can occur simultaneously in elderly patients. It highlights the importance of screening for ATTRwt amyloidosis in patients with heart failure and a history of multiple orthopedic surgeries. Further systematic studies are needed to determine the incidence of dual amyloidosis.
